# Native American Women's Willingness to Screen for Both Cervical and Colorectal Cancer at Home

**DOI:** 10.1002/cam4.71654

**Published:** 2026-03-04

**Authors:** Keely K. Ulmer, Michele D. Sargent, Kristin R. Cina, Alexandra H. Vinson, Timothy C. Guetterman, Daniel G. Petereit, Diane M. Harper

**Affiliations:** ^1^ Cancer Care Institute at Monument Health/Avera Research Institute Rapid City South Dakota USA; ^2^ Walking Forward/Avera Research Institute Rapid City South Dakota USA; ^3^ Department of Learning Health Sciences and Psychiatry University of Michigan Ann Arbor Michigan USA; ^4^ Department of Family Medicine University of Michigan Ann Arbor Michigan USA; ^5^ Department of Family Medicine, Obstetrics & Gynecology, Women's and Gender Studies, and Bioengineering University of Michigan Ann Arbor Michigan USA

**Keywords:** at‐home colorectal cancer screening tests, cancer prevention, cervical cancer screening, HPV testing, native American, self‐sampling

## Abstract

**Introduction:**

Fewer than 50% of Native American (NA) women screen for *both* cervical and colorectal (CRC) cancer. We aim to explore the perspectives of NAs around cervical and colorectal cancer home‐based self‐screening options.

**Methods:**

The NA community provided review and approval for this cross‐sectional survey on cancers in general, and specifically on cervical and colorectal cancer screening. We invited screen‐eligible Native American women, aged 45–65 years, who attended the Lakota Nation Invitational tournament in December 2023, to complete the survey.

**Results:**

One hundred women, with a mean age of 54.1 (SD 6.3), completed the survey. Respondents reported visiting their doctor once a year, rarely (10%), with 66% experiencing a poor experience accessing healthcare—only 16% self‐reported screening for both cervical and colorectal cancers within the last 5 years. If the participant could screen for *both* cervical and CRC cancer at home, 83.0% said they would be willing to do both, compared to 9% who would do neither at home. The doctor's recommendation for how to screen for cervical and CRC cancer was the most important factor in screening decision‐making. The other two very important reasons were how easy or convenient the screening is, how comfortable I am with the screening process/what happens to me during the test.

**Conclusions:**

With the recommendation of their doctors, and convenience and comfort being important, Native American women are enthusiastic to participate in home‐based cervical and colorectal cancer screening. While the home‐based CRC screening has been available for many years, with minimal effect on screening uptake, the advent of self‐sampling for primary HPV testing for cervical cancer appears to create interest for both tests at home. These options may increase both cancer screening rates and access to care in this underserved population.

## 1 Introduction

Women in the US screen the least for cervical and colorectal cancer (CRC) among all screen‐eligible women compared to other cancer screenings. The up‐to‐date screening rate for both cancers is 58%, with 21% up to date with only cervical cancer screening and 12% up to date with only CRC cancer screening [1, 2].

The Anderson Behavior Model of Healthcare Utilization shows that barriers to cervical and colorectal cancer screening include predisposing factors [demographics (age, BMI), social (race/ethnicity, education, occupation, zip code)], enabling factors [organizational (physician type, office hours, medical guideline changes); facility (regular source of care, primary care physician (PCP), travel abilities); and finance (income, health insurance, cost‐sharing)] and needs factors [evaluated (abnormal screening result, severity of abnormality); perceived (sense of general health)] [1, 2].

Native American (NA) history of high cancer incidence. Cancer of any type is the leading cause of death for Native American women [3]. Cervical cancer incidence among NA women in the US is 9.2/100,000 and 45.5/100,000 for CRC, reported in the 2017–2019 SEER data; 33% and 40% higher than the Non‐Hispanic White females, whose incidences are 6.9/100,000 and 32.4/100,000, respectively [4]. A persistently elevated incidence of both cancers has been reported among NAs over the past 30 years [5, 6, 7], despite randomized controlled trials not successfully increasing screening rates [8, 9, 10, 11].

Specific types of self‐collected cancer screening are equally as accurate as invasive exams. Cervical cancer screening using primary high‐risk human papillomavirus (HPV) testing is now recommended by the United States Preventive Services Task Force (USPSTF) and the American Cancer Society (ACS) [12, 13] Many European countries, such as Sweden, Denmark, Germany, and the Netherlands, as well as Australia, have transitioned to primary HPV screening as their national screening program [14] Self‐sampling for HPV testing has been discussed in the literature since 1990 [15, 16, 17, 18, 19]. It has continued to evolve to include both urine and vaginal collection choices [18, 20], with vaginal swabs approved by the US Food and Drug Administration (FDA) for clinical use with an FDA‐approved HPV testing platform [21].

Like the home‐based CRC screening methods, primary HPV screening with self‐collected and home‐based sampling techniques has the potential to reach many women who currently face barriers to traditional speculum‐based screening [22, 23, 24, 25, 26].

Colonoscopy is often perceived as an invasive procedure that requires days of fasting and diarrhea to be adequately prepped. More often, the biggest barriers to CRC screening are having insurance coverage to cover the costs of the procedure, having transportation to the procedure, and needing a person to accompany them [27]. In medical decision making, the harms of the screen cannot be excessive; colonoscopy can cause bleeding and perforation, which can cause significant morbidity, often to those whose screens were normal [28]. Cologuard and fecal immunochemical testing (FIT), two home‐based colorectal cancer screening tests, have long been proven to be non‐inferior to colonoscopy, requiring colonoscopy only for those who test positive [29]. FIT testing, in particular, is widely used in US safety net clinics [30] as a cost‐effective approach, saving around 80% of the population from potential procedural harm [29, 31, 32].

### 1.1 Objectives

We aim to explore the perspectives of community‐based Native Americans about cervical and colorectal cancer home‐based self‐screening options.

## 2 Methods

This is a cross‐sectional study. Regardless of symptoms, we define a screening test for cancer as the first test a woman receives, before determining whether further diagnosis is needed. All participants provided verbal consent.

### 2.1 Population

The Lakota Nation Invitational (LNI) is held annually for 5 days in South Dakota during the winter. It offers an energetic gathering place for multi‐sport tournaments for schools from Indian Reservations in South Dakota, North Dakota, Nebraska, and Wyoming. Native Americans of all ages from across the US attend in the thousands. The LNI has been a valuable gathering for survey research, with active participation at the adjoining booths [33]. Several Native American volunteers canvassed the crowd to inform attendees of the health survey opportunity. Only people who had a cervix, were not pregnant, were between 45 and 65 years of age, did not have a colostomy or hysterectomy, and had not had cervical or CRC cancer, or a family member with CRC cancer, were invited to complete the survey.

### 2.2 Survey Development

Our survey study was exempt on August 20, 2021, by IRB HUM00204087. The survey was developed from the National Cancer Institute's (NCI) Health Information National Trends Survey 5, Cycle 4 (HINTS 5, Cycle 4), created by behavioral researchers [34]. It regularly collects nationally representative data on US knowledge, attitudes, and use of cancer prevention opportunities. Specifically, HINTS measures how people access and use health information to manage their health, as well as the degree to which they are engaged in healthy behaviors, including annual physical exams and activities related to cancer prevention and control.

Specifically, we used question content from nine HINTS modules to create a survey taking less than 15 min to complete: cancer communication, cancer perceptions and knowledge, cervical cancer, CRC cancer, demographics, health communication, health services, health status, and patient‐provider communication. The community planning group (led by M.D.S., K.R.C., K.K.U.) provided feedback on the pilot questions, highlighting topics important to them, as well as topics that were not included and questions that were included but deemed offensive (Appendix S1). The final survey reflected community input. An experienced research nurse trained the Native American volunteers staffing the booth to ensure consistency in survey completion. The training included answering questions about the meaning of the survey questions and providing assistance with completing the survey in a private space, if desired.

### 2.3 Survey Dissemination

Community team members (K.R.C., K.K.U., M.D.S.) hosted a booth where LNI visitors could complete the questionnaire in a quiet, alone space and receive their $20 compensation. Some participants required help to understand the meaning of the survey questions.

All surveys were answered on paper, and the responses were entered into the REDCap database. Double‐entry data checks were performed intermittently.

We included another social determinant measure, the Area Deprivation Index (ADI), which is a calculated value of neighborhood disadvantage. The ADI measure may influence a person's health independent of their income, education, and occupation. The ADI (ranging from 1 (least deprived) to 100 (most deprived)) is created from 17 measures of education, employment, housing quality, and poverty, originally drawn from the long‐form Census data and updated annually with American Community Survey data. It cross‐references over 69 million nine‐digit zip codes that are linked to neighborhood disadvantage [35].

### 2.4 Statistics

We used descriptive statistics, including central tendency measures such as means, standard deviations, frequencies, and confidence intervals, to characterize the population based on its demographic data. We used inferential statistics, including *t*‐tests, analysis of variance (ANOVA), chi‐square tests, and Kruskal‐Wallis multiple means testing, with a two‐sided alpha of 0.05 and Bonferroni corrections for multiple comparisons. The inferential statistics were used to evaluate differences in women's perceptions of the health factors associated with cervical and colorectal cancer screening [36].

## 3 Results

### 3.1 Descriptors of the Population

From all attendees at LNI, one hundred women, with an average age of 54.1 (SD 6.3), completed the survey. 97% were Native American or of mixed race with a White or non‐Hispanic ethnicity. Most (90%) self‐reported having very good or good health, but very few (10%) visited their primary care physician (PCP) once a year for a check‐up, and 66% did not have a good experience accessing healthcare (Table 1).

**TABLE 1:** Descriptors of the population.

Scale ranking	Age, years	*n* (%)	Mean (SD)
	45–65		54.1 (6.3)
Race, *N* = 100			
	Native American/Non‐Hispanic	90 (90)	
	Native American/Hispanic	6 (6)	
	Native American White	1 (1)	
	Hispanic	1 (1)	
	White/Non‐Hispanic	2 (2)	
General health status, *N* = 99			2.34 (0.64)
1	Excellent	9 (9)	
2	Very Good	47 (47)	
3	Good	43 (43)	
4	Fair	0	
5	Poor	0	
I visit my doctor at least once a year for a check‐up, *N* = 97			4.03 (1.00)
1	Strongly agree	1 (1)	
2	Agree	9 (9)	
3	Somewhat agree	14 (14)	
4	Disagree	35 (36)	
5	Strongly disagree	38 (39)	
My experience accessing health care (e.g., going to the doctor) has been mostly positive, *N* = 100			3.62 (1.05)
1	Strongly agree	7 (7)	
2	Agree	6 (6)	
3	Somewhat agree	21 (21)	
4	Disagree	50 (50)	
5	Strongly disagree	16 (16)	
I participate in other health screening programs through an employer or health department that are not my regular doctor, *N* = 99			3.44 (1.23)
1	Strongly agree	9 (9)	
2	Agree	15 (15)	
3	Somewhat agree	19 (19)	
4	Disagree	37 (37)	
5	Strongly disagree	20 (20)	
Current income, *N* = 100			1.77 (0.83)
1	Living comfortably on current income	43 (43)	
2	Getting by with current income	42 (42)	
3	Finding it difficult with the current income	10 (10)	
4	Finding it very difficult with my current income	5 (5)	
Health insurance, *N* = 100			
	Indian Health Service	39 (39)	
	Employer/Personal Purchase	40 (40)	
	Federal (Medicaid/Medicare/Tricare)	14 (14)	
	None	7 (7)	
Education, *N* = 100			
	Less than HS	7 (7)	
	HS or GED graduate	18 (18)	
	Some college	25 (25)	
	Graduated from college (associate's or bachelor's degree)	39 (39)	
	Post‐graduate degree (master's or doctorate)	11 (11)	
National Area Deprivation Index^a^ (1 is least deprived, 100 is most deprived), *N* = 98			
	1–50	5 (5)	
	51–75	16 (16)	
	75–90	6 (6)	
	90–95	6 (6)	
	95–100	65 (66)	
Relationship Status, *N* = 98			
	Married or partnered	45 (46)	
	Single, never married or partnered	25 (26)	
	Widowed/Divorced/Separated	28 (28)	
Disability, *N* = 100			
	No	87 (87)	
	Yes	13 (13)	

^a^ Kind AJH, Buckingham W. Making Neighborhood Disadvantage Metrics Accessible: The Neighborhood Atlas. *New England Journal of Medicine*, 2018; 378: 2456–2458. DOI: 10.1056/NEJMp1802313. PMCID: PMC6051533.

A quarter (24%) participates in health screening programs offered by an employer or other place besides the PCP's office. Most were comfortable with their income (85%), and all but 7% had health insurance. Most (75%) had some college education or more, but 95% lived in zip codes with the worst area deprivation index (ADI) scores. 72% were single or partnered, and 13% identified with a disability.

### 3.2 Cancer Screening Information

Past screenings for any cancer type were common (81%), with mammography and in‐office cervical cancer screening being the most prevalent (Table 2). All who paired cervical and CRC cancer screenings (16%) also screened for breast cancer. Most (94%) had no history of cancer. On a Likert scale of 1–5 (1 very important, 5 not at all important), the most important factor in the decision to screen was the doctor's recommendation (mean 1.24 (0.60)) and knowing the screening options were accurate (1.39 (0.77), NS). Two closely following reasons were how easy or convenient the screening is (1.45 (0.75)), and how comfortable I am with the screening process/what happens to me during the test (1.49 (0.85)). The frequency of screening, national guidelines, insurance company recommendations, out‐of‐pocket costs, and what family members thought were significantly less important than the doctor's recommendation, as well as convenience and comfort with the screening method (*p* < 0.001).

**TABLE 2:** Population descriptors of cancer screening.

	*N* (%)
Have you ever been screened for any cancer?	
Yes	81 (81.0)
No	16 (16.0)
Unsure	3 (3.0)
What types of cancers do you screen for?	
Breast	68 (68.0)
Lung	5 (5.0)
Skin	8 (8.0)
Cervical	51 (51.0)
Colorectal	24 (24.0)
Prior diagnosis of any cancer?	
Yes (1 skin, 2 breast)	3 (3.0)
No	94 (94.0)
Unsure	2 (2.0)
Importance of factors in the decision to screen for any cancer^a^	mean (SD)
Doctor's recommendation on how to get screened for cancer^b^	1.24 (0.60)
How accurate is the screening method	1.39 (0.77)
How easy or convenient the screening is	1.45 (0.75)
How comfortable am I with the screening process/what happens to me during the test	1.49 (0.85)
How often do I need to get screened	1.50 (0.79)
National cancer screening guidelines (CDC)	1.71 (0.94)
Recommendations from cancer advocacy groups (American Cancer Society)	1.76 (0.90)
Out‐of‐pocket costs	1.84 (1.19)
Information from my insurance company	1.93 (1.16)
What do my family or friends think I should do^c^	2.36 (1.30)
Barriers preventing screening	*N* (%)
None	42 (42.0)
One	39 (39.0)
Two	10 (10.0)
Three or more	9 (9.0)
Barriers	
Single	*N*
No time to go to the doctor	9
Not knowing when or if I needed to be screened	8
Uncomfortable with the screening procedure	6
Not having health insurance	4
No transportation	4
Afraid of the results	3
Other	5
Two	
No time to go to the doctor	6
Uncomfortable with the screening procedure	6
Not having health insurance	3
Afraid of the results	3
Not knowing when or if I needed to be screened	1
No transportation	1
Three or more	
Afraid of the results	8
No time to go to the doctor	7
Not knowing when or if I needed to be screened	6
Not having health insurance	3
No transportation	3
Uncomfortable with the screening procedure	3
Other	5
Write in reasons	
Not knowing why I needed to be screened	2
Not enough appointment times are available at the clinic	2
No confidentiality at the clinic	2
No symptoms	1
Screening is not offered at the hospital	1

*Note:* Factors are listed from most important to least important. “The doctor's recommendation to screen” is ranked significantly more important than all other reasons, except for the accuracy of the test (*p* < 0.05). this is what footnote c is supposed to say.

^a^ 1 = very important 2‐somewhat important 3‐neutral 4‐not very important 5 = not at all important.

^b^ A screening test is performed on both symptomatic and asymptomatic women as the initial test before further diagnostic workup.

^c^ “What my friends and family think I should do” is ranked significantly less important than every other reason.

### 3.3 Barriers

42% of participants reported no barriers to any cancer screening. 39% reported a single barrier, 10% reported two barriers, and 9% reported three or more barriers preventing screening. “No time to go to the doctor” was the most frequent barrier (a single barrier in nine women, one of two barriers among six women, and one of three or more barriers for seven women). “Not knowing when or if I needed to be screened” tied with “Uncomfortable with the screening procedures,” where “*not knowing when or if I needed to be screened*” occurred as a single barrier in eight women, one of two barriers in one woman, and one of three or more barriers among six women. “*Uncomfortable with screening procedures*” was a single barrier among six women, one of two barriers in six women, and one of three or more barriers in three women. “Afraid of the results” was the next most common barrier, occurring as a single barrier for three women, one of two barriers for three women, and one of three or more barriers for eight women. The least frequent barrier was “no transportation,” occurring as a single barrier among four women, as one of two barriers for one woman, and as one of three or more barriers for three women.

### 3.4 Comparing Cervical and Colorectal Cancer Screening

On a Likert scale of 1–4 (1 = very important, 4 = not important at all), respondents indicated that both cervical and CRC cancer screening were very important (1.07 (0.26) vs. 1.15 (0.44), *p* = 0.52) (Table 3). Significantly more women have had their doctor discuss cervical cancer screening with them than CRC cancer screening (81.8% vs. 63.0%, *p* < 0.01); and significantly more have been screened for cervical cancer than for CRC cancer (78.0% vs. 45.0%, *p* < 0.001).

**TABLE 3:** Comparisons of characteristics of cervical and colon cancer screening.

	Cervical cancer	Colon cancer	*p*‐value
Importance of screening^a^, mean (SD)	1.07 (0.26)	1.15 (0.44)	NS
Has your doctor talked with you about…, *N* (%)			< 0.01
Yes	81 (81.8)	63 (63.0)	
No	15 (15.2)	33 (33.0)	
Unsure	3 (3.0)	4 (4.0)	
Ever screened for…, *N* (%)			< 0.001
Yes	78 (78.0)	45 (45.0)	
No	17 (17.0)	49 (49.0)	
Unsure	5 (5.0)	6 (6.0)	
When was the last time you were screened, *N* (%)			< 0.001
Less than 3 years	52 (66.7)		
3–5 years^d^	16 (20.5)		
6–10 years	10 (12.8)		
More than 10 years	0 (0.0)		
Less than 1 year		9 (21.4)	
Between 1 and 5 years^e^		22 (52.4)	
Between 6 and 10 years		9 (21.4)	
More than 10 years		2 (4.8)	
Method of screening, *N* (%)			
Cervical Cytology	54 (71.1)		
HPV test	0 (0.0)		
Co‐test (both cytology and HPV)	20 (26.3)		
Unsure	2 (2.6)		
Colonoscopy		29 (53.7)	
Stool test (Cologuard, FIT, FOBT)		22 (40.7)	
Unsure		3 (5.6)	
Comfort of the test you used^b^, mean (SD)			
Speculum exam	3.37 (1.20)		
Colonoscopy		3.31 (1.47)	
Stool test		3.59 (0.85)	
Social Perceptions of the Screening Process^c^			
Some perception, mean (SD)	2.59 (0.89)	2.89 (0.93)	
No perception, *N* (%)	33 (34.0)	29 (29.0)	

*Note:* Frequency comparisons were analyzed with chi‐square testing. Scale comparisons were analyzed with *t*‐tests. NS means *p* > 0.05.

^a^ 1 = very important 2 = somewhat important 3 = only a little important 4 = not important at all.

^b^ 1 = very comfortable 2 = mostly comfortable 3 = neutral 4 = mostly uncomfortable 5 = very uncomfortable.

^c^ 1 = very positive 2 = mostly positive 3 = neutral 4 = mostly negative 5 = very negative.

^d^ The guidelines recommended screening interval.

^e^ The guidelines recommended screening interval.

Only 20.5% self‐reported their last cervical cancer screening in the 3–5 year time frame, per guideline recommendations. The most common cervical cancer screening technique was cytology (71.1%), followed by HPV‐based co‐testing (26.3%). The most common time frame for CRC cancer screening was reported to be between 1 and 5 years (52.4%), which aligns with guideline recommendations. Colonoscopy (53.7%) and home‐based stool tests (40.7%) were equally reported as the most common techniques for CRC cancer screening.

The reported comfort with the in‐office techniques was assessed on a 1–5 Likert scale (1 = very comfortable, 5 = very uncomfortable), with a rating of 5 indicating great discomfort. Both the speculum exam and the colonoscopy were ranked in the discomfort half (3.37 (1.20) vs. 3.31 (1.47), NS, respectively).

### 3.5 Preferences for Potential Self‐Collection

Preferences for potential self‐collection at home for cervical and CRC cancer differed (Table 4). On a 1–4 Likert scale (1 very comfortable, 4 very uncomfortable), the urine collection concept was significantly more favored than the vaginal (1.30 (0.68) vs. 1.73 (0.98), *p* < 0.01). The home stool collection was favored less than the HPV collection devices (2.35 (0.65), *p* < 0.001), but still had a positive perception. When asked which potential home‐based cervical cancer screening device was preferred, there were equal responses for urine and either vaginal or urine (50.0% vs. 43.0%, NS), with only 2% indicating neither home‐based screen.

**TABLE 4:** Preferences about self‐collection at home for cervical and colon cancer screening.

	Cervical cancer	Colon cancer
Comfort collecting sample at home^a^, mean (SD)	Urine: 1.30 (0.68)	Stool: 2.35 (0.65)**
	Vaginal: 1.73 (0.98)*	
Which home‐based cervical cancer screening test do you prefer? *N* (%)		
Vaginal	4 (4.0)	
Urine	50 (50.0)	
Either	43 (43.0)	
Neither	2 (2.0)	
Willingness to discuss home sampling, *N* (%)		
Yes	70 (70.0)	79 (79.0)
Maybe	21 (21.0)	11 (11.0)
No	9 (9.0)	9 (9.0)
Choice of how to be screened, when due, *N* (%)		
Coming to the clinic for a speculum exam ǁ colonoscopy	47 (47.0)	38 (38.0)
Receiving a home kit (urine or vag) ǁ (stool) to use and return	43 (43.0)	50 (50.0)
Rather not be screened at all	3 (3.0)	3 (3.0)
Unsure	7 (7.0)	8 (8.0)
The likelihood of screening with a home option, *N* (%)		
More Likely	78 (78.0)***	70 (70.0)***
Less likely	5 (5.0)	11 (11.0)
Not changing my likelihood of being screened	14 (14.0)	19 (19.0)
Encourage others to use the screen at home options^b^, mean (SD)	1.64 (1.09)	1.89 (1.24)

^a^ 1 = very comfortable 2 = somewhat comfortable 3 = uncomfortable 4 = very uncomfortable.

^b^ 1 = very likely 2 = moderately likely 3 = somewhat likely 4 = not very likely 5 = not likely at all.

* *p* < 0.01 compared to urine.

** *p* < 0.001 compared to urine or vaginal.

*** *p* < 0.0001 more likely compared to not changing my likelihood of being screened.

Women indicated that they would be more likely to screen for cervical and CRC cancer if there were a home option, significantly more than those who said it would not change their likelihood of screening (78.0% vs. 14.0%, *p* < 0.0001, and 70.0% vs. 19.0%, *p* < 0.0001, respectively). On a Likert scale of 1–5 (1 very likely, 5 not likely at all), the participants were likely to encourage others to use the cervical and CRC home‐based screenings (1.64 (1.09) and 1.89 (1.24), NS).

### 3.6 Women's Future Screening Intentions

In the future case where the participant could screen for both cervical and CRC cancer, 83.0% said they would be willing to do both at home, compared to 9% who would do neither at home, or only do a single type of test (cervical only 5.0%, CRC only 3.0%) (Table 5). Neither age, ADI, health status, poor experience accessing health care, income, education, health insurance, relationship, nor disability status was associated with the likelihood of choosing to do both self‐screenings at home.

**TABLE 5:** If your doctor told you that you were currently due for BOTH cervical and colon cancer screening.

	*N* (%)
Which home tests would you be willing to do?	
Cervical cancer only	5 (5.0)
Colon cancer only	3 (3.0)
Both	83 (83.0)
Neither	9 (9.0)
If you were already doing cervical cancer screening at home, how likely would you be to try the colon cancer home screen if you were sent both screens at the same time?^a^ mean (SD)	1.80 (0.84)
If you were already doing colon cancer screening at home, how likely would you be to try the cervical cancer home screen if you were sent both screens at the same time?^a^ mean (SD)	1.67 (0.58)
Would you want to receive both cervical and colon cancer screening kits on the same day^a^	
Both on the same day	66 (88.0)
Separately	9 (12.0)
What might encourage you to try home‐based cancer screening in the future?^b^	
Recommendation from my doctor	46
Learning more about at‐home screening	37
Talking to someone who has already done it	30
If my partner/spouse also used home‐based cancer screens	11
Receiving information from my insurance company	7
Nothing	5
Other	2

^a^ 1 = very likely 2 = moderately likely 3 = somewhat likely 4 = not very likely 5 = not likely at all.

^b^ More than one option can be chosen.

On a Likert scale from 1 (very likely) to 5 (not likely at all), if the woman was already using a home‐based stool test for CRC cancer, she was also highly likely to use a home‐based cervical cancer screening test (1.67, 0.58). 88.0% would prefer to receive both cervical and CRC home cancer screening tests in the mail simultaneously, compared to 12.0% who preferred two separate screening events.

The most important encouragement for future home‐based cancer screenings was the doctor's recommendation, followed by additional education about the home‐based tests, including testimonials on how to perform them. The least influential factor was the information provided by insurance companies.

## 4 Discussion

Community team members participated in the interpretation of all results, leading to our interpretation that the Native American women's likelihood of home‐based screening for *both* cervical and colorectal cancers is high; both cancer screens have FDA‐approved self‐screening techniques for each respective cancer in the US. Our work among Native Americans indicated that 83% would use *both* screening tests at home, up from the 16% who had previously screened for both in the office. Because of the persistently higher rates of cervical and CRC cancers among Native Americans, home‐based screenings may offer an advantage for cancer prevention.

Up‐to‐date independent cervical and CRC cancer screening rates vary widely in the US, among women aged 50–74, with a difference of at least 10 points (e.g., 75% vs. 65%, with cervix screening more prevalent than CRC screening) [1, 37, 38]. In our current work, 51% and 24% of Native American women reported having ever been screened for cervical or CRC cancer, supporting the deficit in both cancer screenings historically seen [39, 40], as well as the lack of change in low screening rates over time [10].

Native Americans have screening rates more closely aligned with global populations, where the speculum‐based cervical cancer screening occurs more frequently than the only available test of at‐home CRC cancer screening [41, 42, 43]. Outside the US, many global studies report less than 5% of women completing *both* a cervical and CRC cancer screening test [41, 42, 43]. The Danish registry offers hope for increased uptake, where 56% of screening‐eligible women have had *both* cervical and CRC cancer screening [44], where the stool test was done at home. Since 2015, when the FDA approved the multi‐target stool (mt‐stool) test for CRC screening, the uptake of home‐based testing increased from zero to 15.3% through 2021 [45].

Prior experience with cancer screens can be helpful when introducing home‐based screening tests. A study of women from Scotland who participated in office‐based cervical cancer screening had a threefold increased odds of completing a home‐based CRC cancer screening test [41]. Taking the next step, our data showed that those who screened with an at‐home CRC cancer test are also more likely to complete the home‐based cervical cancer screen.

Other literature suggests that wordless instructions accompanied by the same skin‐toned images of the population serve to facilitate the use of home‐based cancer screening [46, 47]. Additionally, other literature suggests that women prefer to use the self‐screening test in the office to avoid potential implementation issues [48, 49].

In our study, Native American women reported being very comfortable with both home‐based cervical cancer and colon cancer screening, and about half preferred to use and return both cervical and colon cancer screening tests. The literature reports discomfort, embarrassment, pain, and worry about the test result for colorectal cancer screening reported by women for only the colonoscopy test, not the home‐based test options; and this discomfort was a barrier to screening for 29% of women [49]. Other literature reports comfort with urine and vaginal cervical cancer screening among different racial/ethnic populations, who report a high likelihood of self‐sampling once their doctor's office offers the option [22, 24, 48].

Our work showed that the doctor's recommendation for screening for cervical and CRC cancer was the most important factor in screening decision‐making. Our results align with the increased intent to screen and actual screening reported in other studies [38, 50]. Our population reports similar numbers of barriers to cervical and CRC cancer screening as other non‐Native populations. Similar to our research, in the UK, among women who actively decline screening, 86% of women had one barrier preventing them from cervical cancer screening, 9% had two, and 5% had three barriers. Finding time for screening and being frightened of the results were similarly common barriers [51]. Our work also demonstrates that NA women highly value the convenience and comfort of a screening test, as observed in previous studies [22, 23, 24, 52].

### 4.1 Strengths and Limitations

Most NA research is limited by the comparatively small NA population within the US (3–8 million), high heterogeneity among states and tribes, geographic dispersion, and inadequate representation in the existing survey data. Tribal populations range from 30 in Lime City, AL, to 400,000 in the Cherokee Nation in Oklahoma, and from 2000 in Vermont to 1.1 million in California [53, 54, 55]. Our strength lies in conducting an in‐person survey of a large gathering of Native Americans from across the US attending LNI. Our most powerful strength is the co‐design of the study with community partners. The community partners enabled a space at LNI for the survey and collected all data elements.

### 4.2 Limitations

Our study population is more educated (graduated from some college: 50% vs. 14%), in better health (56% vs. 42%), less often never married (26% vs. 33%), and less disabled (13% vs. 24%) than the population described by the American Community Survey of Native Americans [56, 57] but has a similar distribution of health insurance/access sources. Although the population was more educated, there were likely some women who did not understand the questions and did not ask for help in interpreting them. Another limitation of our study is that it is purely quantitative. A future need is for qualitative research to understand the reasons why women make their decisions.

## 5 Conclusions

With the recommendation of their doctors and the convenience and comfort of self‐sampling, Native American women are willing to participate in home‐based cervical and colorectal cancer screening. While the home‐based CRC cancer screening has been available for many years, with minimal effect on screening uptake, the advent of self‐sampling for primary HPV testing for cervical cancer appears to create enthusiasm for both tests at home. Our future research will test the uptake of both cervical and colorectal cancer screening among NAs at their preferred location, either at home or at the office. These options may increase both cancer screening rates and access to care in this underserved population.

## Author Contributions

All authors take full responsibility for this article.

## Funding

This study was supported by the American Cancer Society, PASD‐RSG‐23‐1077156‐01‐PASD. National Cancer Institute, P30CA046592.

## Disclosure

16% of Native American women report both cervical and colorectal cancer screening completion. 83% of Native American women would screen for both cancers if the screening were offered as home‐collected options.

## Conflicts of Interest

The authors declare no conflicts of interest.

## Supporting information


**Appendix S1:** cam471654‐sup‐0001‐Apeendix.pdf.

## Data Availability

The data that support the findings of this study are available on request from the corresponding author. The data are not publicly available due to privacy or ethical restrictions.
