# Supplementary material for: Native American Women's Willingness to Screen for Both Cervical and Colorectal Cancer at Home
Source: Cancer Med. 2026 Mar 4;15(3):e71654. doi: 10.1002/cam4.71654 (PMC12961161; doi:10.1002/cam4.71654)
Supplement: Supplementary file 1 — Appendix S1: cam471654‐sup‐0001‐Apeendix.pdf. [file CAM4-15-e71654-s001.pdf]

Data Dictionary Codebook

12/07/2022 2:24pm

	#	Variable / Field Name	Field Label<br>*Field Note*	Field Attributes (Field Type, Validation, Choices, Calculations, etc.)
**Instrument: English Survey (english_survey) Enabled as survey**				
	1	record_id	Record ID	text
	2	health_text	Section Header: *General Health*<br>In this first section, we will ask you some questions about your current health and healthcare practices	descriptive
	3	health	In general, would you say your health is:	radio<br>1 Excellent<br>2 Very good<br>3 Good<br>4 Fair<br>5 Poor
	4	age	How old are you?<br>**You are not eligible if you are under 45 or over 65*	dropdown (autocomplete), Required<br>1 Younger than 45<br>2 45<br>3 46<br>4 47<br>5 48<br>6 49<br>7 50<br>8 51<br>9 52<br>10 53<br>11 54<br>12 55<br>13 56<br>14 57<br>15 58<br>16 59<br>17 60<br>18 61<br>19 62<br>20 63<br>21 64<br>22 65<br>23 Older than 65<br><br>Stop actions on 1, 23
	5	sex	Which of the following best characterizes your body in terms of biological sex?	radio, Required<br>1 Female

			You are not eligible if you are a male	2 Male<br>3 Non-binary<br><br>Stop actions on 2
	6	cervix<br><br>Show the field ONLY if:<br>[sex] = '3'	Do you have a cervix?<br>*You are not eligible if you answer no*	radio, Identifier<br>1 Yes<br>2 No<br><br>Stop actions on 2
	7	hysterectomy	Have you had a hysterectomy? (surgery to remove your uterus)<br>*You are not eligible if you answer yes*	radio, Required<br>1 Yes<br>2 No<br><br>Stop actions on 1
	8	colostomy	Do you have a colostomy? (An opening in the belly made by a doctor as a way for bowel movements to leave the body)<br>*You are not eligible if you answer yes*	radio, Required<br>1 Yes<br>2 No<br><br>Stop actions on 1
	9	family_hx_colon	Do you have a family history of colon cancer?<br>*You are not eligible if you answer yes*	radio, Required<br>1 Yes<br>2 No<br><br>Stop actions on 1
	10	colon_adenoma_hx	Have you yourself ever been diagnosed with a colon adenoma?<br>*You are not eligible if you answer yes*	radio, Required<br>1 Yes<br>2 No<br><br>Stop actions on 1
	11	text	Indicate the extent to which you agree with the following statements:	descriptive
	12	primarycare	I visit my doctor at least once a year for a physical, check-up, or recommended health screening	radio<br>1 Strongly agree<br>2 Agree<br>3 Somewhat agree<br>4 Disagree<br>5 Strongly disagree
	13	primarycare_2	I participate in other health screening programs through an employer, health department, or other facilities that are not my regular doctor	radio<br>1 Strongly agree<br>2 Agree<br>3 Somewhat agree<br>4 Disagree<br>5 Strongly disagree
	14	postive	My experiences accessing healthcare (e.g., going to the doctor) have been mostly positive	radio<br>1 Strongly agree<br>2 Agree<br>3 Somewhat agree

			4 Disagree<br>5 Strongly disagree
15	page1		descriptive
16	screen_text	Section Header: *Cancer Screening*<br>This next set of questions will ask you about your experiences with cancer screening	descriptive
17	screen_hx	Have you ever been screened for any kind of cancer before? (e.g. breast, lung, cervical, skin, colon)	radio<br>1 Yes<br>2 No<br>3 Unsure / I don't know
18	screen_hx_type<br>Show the field ONLY if:<br>[screen_hx] = '1'	What type(s) of cancer have you been screened for? [mark all that apply]	checkbox<br>1 screen_hx_type___1 Breast cancer<br>2 screen_hx_type___2 Lung cancer<br>3 screen_hx_type___3 Skin cancer<br>4 screen_hx_type___4 Cervical cancer<br>5 screen_hx_type___5 Colon cancer<br>6 screen_hx_type___6 Other type(s) of cancer (list below)
19	screen_hx_other<br>Show the field ONLY if:<br>[screen_hx_type(6)] = '1'	Other type(s) of cancers:	text
20	dx_hx	Have you ever been diagnosed with any kind of cancer before?	radio<br>1 Yes<br>2 No<br>3 Unsure / I don't know
21	dx_hx_type<br>Show the field ONLY if:<br>[dx_hx] = '1'	What type(s) of cancer have you been diagnosed with? [mark all that apply]	checkbox<br>1 dx_hx_type___1 Breast cancer<br>2 dx_hx_type___2 Lung cancer<br>3 dx_hx_type___3 Skin cancer<br>4 dx_hx_type___4 Cervical cancer<br>5 dx_hx_type___5 Colon cancer<br>6 dx_hx_type___6 Other type(s) of cancer (list below)
22	dx_hx_other<br>Show the field ONLY if:<br>[dx_hx_type(6)] = '1'	Other type(s) of cancers:	text
23	prevent_screen	Have any of the following ever prevented you from getting screened for cancer? [mark all that apply]	checkbox<br>1 prevent_screen___1 Not having health insurance<br>2 prevent_screen___2 Not knowing when or if I needed to be screened<br>3 prevent_screen___3 Having trouble finding the time to go to the doctor<br>4 prevent_screen___4 Not having transportation to get to my appointments<br>5 prevent_screen___5 Being uncomfortable with

				(continued) the procedure to get screened (e.g. mammogram, Pap smear, colonoscopy, CT scan)<br>6 prevent_screen___6 Being scared of knowing the results<br>7 prevent_screen___7 Other reason(s) (list below)<br>8 prevent_screen___8 None of the above<br><br>Field Annotation: @NONEOFTHEABOVE='8'
	24	prevent_screen_other2<br><br>Show the field ONLY if:<br>[prevent_screen(7)] = '1'	Other reason(s):	notes
	25	page2		descriptive
	26	cervix_screen_text	Section Header: *Cervical Cancer*<br>The following questions focus specifically on cervical cancer screening. If you are 30-65 years old there are two recommended ways to get screened: 1. Pap smear - A test performed by your doctor that uses a speculum to collect a sample of cells from your cervix during a pelvic exam. Your doctor looks at these cells to see if they show any changes caused by an HPV infection that may be a sign of cervical cancer. 2. HPV test - Uses a vaginal or cervical swab to test for the presence of the Human Papillomavirus (HPV), the virus that causes cervical cancer. HPV is very common and usually goes away on its own, but sometimes an HPV infection can persist for many years and this can lead to cervical cancer. Testing for HPV is a more accurate way to predict cervical cancer risk than a Pap smear. Because of this, you can go longer between screenings with an HPV test compared to a Pap smear. If you test negative for HPV you don't need to be screened again for another 5 years. If you just have a Pap smear and it comes back normal, you will need to screen again in 3 years.	descriptive
	27	screen_prevent_cervix	How important do you think screening is for preventing cervical cancer?	radio<br>1 Very important<br>2 Somewhat important<br>3 Only a little important<br>4 Not important at all
	28	md_talk_cervix	Has your doctor ever talked with you about cervical cancer screening?	radio<br>1 Yes<br>2 No<br>3 Unsure / I don't know
	29	screen_hx_cervix	As far as you know, have you ever been screened for cervical cancer?	radio<br>1 Yes<br>2 No<br>3 Unsure / I don't know
	30	screen_hx_time_cervix<br><br>Show the field ONLY if:<br>[screen_hx_cervix] = '1'	When was the last time you were screened?	radio<br>1 Less than 3 years ago<br>2 Between 3-5 years<br>3 Between 6-10 years

				4 More than 10 years ago
	31	screen_method_cervix<br>Show the field ONLY if:<br>[screen_hx_cervix] = '1'	Do you happen to know what method of screening was used?	radio<br>1 Pap smear<br>2 HPV test<br>3 Co-test (both a Pap smear and an HPV test together)<br>4 Unsure / I don't know
	32	screen_comfort_cervix<br>Show the field ONLY if:<br>[screen_hx_cervix] = '1'	How comfortable was your experience being screened for cervical cancer?	radio<br>1 Very comfortable<br>2 Mostly comfortable<br>3 Neutral<br>4 Mostly uncomfortable<br>5 Very uncomfortable
	33	heard_cervix	What kind of things have you heard from other people about getting screened for cervical cancer?	radio<br>1 Very positive things<br>2 Mostly positive things<br>3 Neutral things (neither positive or negative)<br>4 Mostly negative things<br>5 Very negative things<br>6 NA - I haven't heard anything
	34	page3		descriptive
	35	colon_screen_text	Section Header: *Colon Cancer*<br>The following questions focus specifically on colon cancer screening. If you are 45-75 years old there two recommended ways to get screened: 1. Colonoscopy - A procedure that allows a healthcare provider to view the inside of your colon with a tiny video camera that is inserted into your rectum/anus through a long flexible tube. The day before the appointment you are asked to stop eating solid foods and to drink a liquid that causes bowl clearing diarrhea. 2. Stool Test (Cologuard, FOBT, FIT) - Uses a stool sample (poop) to check for signs of blood or other markers of cancer risk. If you have a positive screen from a stool test it is typically followed up by a colonoscopy. Colonoscopy is a more invasive procedure that requires a day of prep and a trip to the healthcare center. However, if it comes back negative you only need to be screened once every 10 years. Stool tests are less invasive and can be done at home without a procedure or trip to the healthcare center. However, you need to be screened more frequently, once every 1-3 years.	descriptive
	36	screen_prevent_colon	How important do you think screening is for preventing colon cancer?	radio<br>1 Very important<br>2 Somewhat important<br>3 Only a little important<br>4 Not important at all
	37	md_talk_colon	Has your doctor ever talked with you about colon cancer screening?	radio<br>1 Yes<br>2 No<br>3 Unsure / I don't know

	38	screen_hx_colon	As far as you know, have you ever been screened for colon cancer?	radio<br>1 Yes<br>2 No<br>3 Unsure / I don't know
	39	screen_hx_time_colon<br>Show the field ONLY if:<br>[screen_hx_colon] = '1'	When was the last time you were screened?	radio<br>1 Less than 1 year ago<br>2 Between 2-5 years ago<br>3 Between 6-10 years ago<br>4 More than 10 years ago
	40	screen_method_colon<br>Show the field ONLY if:<br>[screen_hx_colon] = '1'	What methods of screening have you done before? [mark all that apply]	checkbox<br>1 screen_method_colon___1 Colonoscopy<br>2 screen_method_colon___2 Stool test (e.g. Cologuard, FOBT, FIT)<br>3 screen_method_colon___3 Unsure / I don't know<br><br>Field Annotation: @NONEOFTHEABOVE='3'
	41	screen_comfort_colonoscopy<br>Show the field ONLY if:<br>[screen_method_colon(1)] = '1'	How comfortable was your experience with a colonoscopy?	radio<br>1 Very comfortable<br>2 Mostly comfortable<br>3 Neutral<br>4 Mostly uncomfortable<br>5 Very uncomfortable
	42	screen_comfort_stool<br>Show the field ONLY if:<br>[screen_method_colon(2)] = '1'	How comfortable was your experience with a stool test?	radio<br>1 Very comfortable<br>2 Mostly comfortable<br>3 Neutral<br>4 Mostly uncomfortable<br>5 Very uncomfortable
	43	screen_comfort_unsure<br>Show the field ONLY if:<br>[screen_method_colon(3)] = '1'	How comfortable was your experience being screened for colon cancer?	radio<br>1 Very comfortable<br>2 Mostly comfortable<br>3 Neutral<br>4 Mostly uncomfortable<br>5 Very uncomfortable
	44	heard_colon	What kind of things have you heard from other people about getting screened for colon cancer?	radio<br>1 Very positive things<br>2 Mostly positive things<br>3 Neutral things (neither positive or negative)<br>4 Mostly negative things<br>5 Very negative things<br>6 NA - I haven't heard anything
	45	page4		descriptive
	46	home_screening_text	Section Header: *Home Based Screening*	descriptive

			Traditionally, getting screened for cancer requires an appointment with your doctor. However, for both colorectal cancer and cervical cancer, there are screening tests you can do at home. For colon cancer there are several stool testing kits available (e.g. Cologuard, FOBT, FIT) that allow you to collect a stool sample (poop) at home and send it through the mail for testing.For cervical cancer there are several HPV testing kits being developed that would allow you to collect a vaginal or urine sample (pee) at home and send it through the mail for testing.	
	47	kits		descriptive
	48	page5		descriptive
	49	md_recommend	Section Header: *When it comes to deciding how you get screened for cancer, how important are the following factors?*<br>What my doctor recommends	radio (Matrix)<br>1 Very important<br>2 Somewhat important<br>3 Neutral<br>4 Not very important<br>5 Not at all important
	50	ease	How easy or convenient the screening is to do	radio (Matrix)<br>1 Very important<br>2 Somewhat important<br>3 Neutral<br>4 Not very important<br>5 Not at all important
	51	frequency	How often I need to get screened	radio (Matrix)<br>1 Very important<br>2 Somewhat important<br>3 Neutral<br>4 Not very important<br>5 Not at all important
	52	comfort	How comfortable I am with how the screening is done and what happens to me during the test	radio (Matrix)<br>1 Very important<br>2 Somewhat important<br>3 Neutral<br>4 Not very important<br>5 Not at all important
	53	accuracy	How accurate the screening method is	radio (Matrix)<br>1 Very important<br>2 Somewhat important<br>3 Neutral<br>4 Not very important<br>5 Not at all important
	54	family	What my family or friends think I should do (e.g., my spouse/partner)	radio (Matrix)<br>1 Very important<br>2 Somewhat important<br>3 Neutral

				4 Not very important<br>5 Not at all important
	55	insurance	Information from my insurance company	radio (Matrix)<br>1 Very important<br>2 Somewhat important<br>3 Neutral<br>4 Not very important<br>5 Not at all important
	56	costs	Out of pocket costs	radio (Matrix)<br>1 Very important<br>2 Somewhat important<br>3 Neutral<br>4 Not very important<br>5 Not at all important
	57	guidelines	National cancer screening guidelines (e.g., from the CDC)	radio (Matrix)<br>1 Very important<br>2 Somewhat important<br>3 Neutral<br>4 Not very important<br>5 Not at all important
	58	advocacy	Recommendations from cancer advocacy groups (e.g., from the American Cancer Foundation)	radio (Matrix)<br>1 Very important<br>2 Somewhat important<br>3 Neutral<br>4 Not very important<br>5 Not at all important
	59	page6		descriptive
	60	home_cervix_text	Section Header: *Home Based Screening: Cervical Cancer*<br>For home-based cervical cancer screening there are two types of samples that can be used. 1. Urine sample - collected by peeing into a cup 2. Vaginal sample - collected by inserting a small swab like a tampon with an applicator Either of these samples can be collected yourself at home and then sent through the mail for testing using a prepaid shipping box.	descriptive
	61	urine_comfort	What would you say your comfort level is for collecting a urine sample (pee) at home? (if you were mailed a collection kit with instructions)	radio<br>1 I would be very comfortable doing this<br>2 I would be somewhat comfortable<br>3 I would be uncomfortable<br>4 I would be very uncomfortable
	62	vaginal_comfort	What would you say your comfort level is for collecting a vaginal sample (swab) at home? (if you were mailed a collection kit with instructions)	radio<br>1 I would be very comfortable doing this<br>2 I would be somewhat comfortable<br>3 I would be uncomfortable<br>4 I would be very uncomfortable

	63	cervix_discuss	If your doctor told you that you were currently due for cervical cancer screening, would you be willing to discuss the option of doing a self-testing kit at home instead of going to the doctor for a Pap smear?	radio<br>1 Yes<br>2 No<br>3 Maybe
	64	cervix_choice	If your doctor told you that you were currently due for cervical cancer screening and you were given the choice of how to be screened, which do you think you would choose?	radio<br>1 Coming into the clinic for a Pap smear and/or HPV test with your doctor<br>2 Receiving an HPV self-testing kit at home and mailing back a sample to be tested<br>3 I would rather not be screened at all<br>4 Unsure / I don't know
	65	cervix_sample	In the future if you had the choice between providing a urine sample or a vaginal sample for the test which would you prefer?	radio<br>1 Urine sample<br>2 Vaginal sample<br>3 Either - I would equally do one or the other<br>4 Neither - I would not do either one
	66	cervix_likelihood	How, if at all, would having the option to use a self-testing kit at home impact your likelihood of getting screened for cervical cancer?	radio<br>1 I would be more likely to get screened<br>2 I would be less likely to get screened<br>3 It would not change my likelihood of being screened
	67	cervix_encourage	If you tried screening for cervical cancer with an HPV test kit at home, how likely would you be to encourage family or friends to try it if they were also due for screening?	radio<br>1 Very likely<br>2 Moderately likely<br>3 Somewhat likely<br>4 Not very likely<br>5 Not likely at all
	68	cervix_encourage_who<br>Show the field ONLY if:<br>[cervix_encourage] = '1' or [cervix_encourage] = '2' or [cervix_encourage] = '3'	Who would you be most likely to encourage? [mark all that apply]	checkbox<br>1 cervix_encourage_who___1 Spouse or partner<br>2 cervix_encourage_who___2 Siblings<br>3 cervix_encourage_who___3 Children<br>4 cervix_encourage_who___4 Parents<br>5 cervix_encourage_who___5 Other family members<br>6 cervix_encourage_who___6 Friends<br>7 cervix_encourage_who___7 Co-workers
	69	home_barriers_cervical	Are there any barriers that you think would make it difficult for you to screen for cervical cancer at home or any things that make you not want to try an HPV home testing kit?	notes
	70	page7		descriptive
	71	home_colon_text	Section Header: *Home Based Screening: Colon Cancer*<br>For home-based colon cancer screening there is one type of sample that can be used: 1. Stool sample - collected by pooping into a container placed underneath your toilet seat that catches the poop before it goes into the toilet This sample can be collected yourself at home and then sent through the mail for testing using a pre-paid shipping	descriptive

			box.	
	72	stool_comfort	What would you say your comfort level is for collecting a stool sample (poop) at home? (if you were mailed a collection kit with instructions)	radio<br>1 I would be very comfortable doing this<br>2 I would be somewhat comfortable<br>3 I would be uncomfortable<br>4 I would be very uncomfortable
	73	colon_discuss	If your doctor told you that you were currently due for colon cancer screening, would you be willing to discuss the option of doing stool test at home instead of going to the doctor for a colonoscopy?	radio<br>1 Yes<br>2 No<br>3 Maybe
	74	colon_choice	If your doctor told you that you were currently due for colon cancer screening and you were given the choice of how to be screened, which do you think you would choose?	radio<br>1 Coming into the clinic for a colonoscopy<br>2 Receiving a stool test kit at home and mailing back a sample to be tested<br>3 I would rather not be screened at all<br>4 Unsure / I don't know
	75	colon_likelihood	How, if at all, would having the option to use a self-testing kit at home impact your likelihood of getting screened for colon cancer?	radio<br>1 I would be more likely to get screened<br>2 I would be less likely to get screened<br>3 It would not change my likelihood of being screened
	76	colon_encourage	If you tried screening for colon cancer with a stool test kit at home, how likely would you be to encourage family or friends to try it if they were also due for screening?	radio<br>1 Very likely<br>2 Moderately likely<br>3 Somewhat likely<br>4 Not very likely<br>5 Not likely at all
	77	colon_exchange_who<br>Show the field ONLY if:<br>[colon_encourage] = '3' or [colon_encourage] = '2' or [colon_encourage] = '1'	Who would you be most likely to encourage? [mark all that apply]	checkbox<br>1 colon_exchange_who___1 Spouse or partner<br>2 colon_exchange_who___2 Siblings<br>3 colon_exchange_who___3 Children<br>4 colon_exchange_who___4 Parents<br>5 colon_exchange_who___5 Other family members<br>6 colon_exchange_who___6 Friends<br>7 colon_exchange_who___7 Co-workers
	78	home_barriers_colon	Are there any barriers that you think would make it difficult for you to screen for colon cancer at home or any things that make you not want to try a home stool testing kit?	notes
	79	page8		descriptive
	80	both_screen_text	Section Header: *Home Based Screening: Both Cancers*<br>Imagine your doctor told you that you were currently due for both cervical and colon cancer screening.	descriptive
	81	screen_choice	If you doctor offered you the option to do these cancer screening tests at home, which would you be open to trying?	radio<br>1 Cervical cancer only

			2 Colon cancer only<br>3 Both<br>4 Neither
82	cervix_colon<br><br>Show the field ONLY if:<br>[screen_choice] = '1'	If you were already doing cervical cancer screening at home, how likely would you be to try the colon cancer screen in the future if you were sent both testing kits at the same time?	radio<br>1 Very likely<br>2 Moderately likely<br>3 Somewhat likely<br>4 Not very likely<br>5 Not likely at all
83	colon_cervix<br><br>Show the field ONLY if:<br>[screen_choice] = '2'	If you were already doing colon cancer screening at home, how likely would you be to try the cervical cancer screen in the future if you were sent both testing kits at the same time?	radio<br>1 Very likely<br>2 Moderately likely<br>3 Somewhat likely<br>4 Not very likely<br>5 Not likely at all
84	both_timing<br><br>Show the field ONLY if:<br>[screen_choice] = '3'	Would you like to receive both cancer screening kits the same day (i.e., one trip to the bathroom to collect both samples) or would you prefer spreading them out throughout the year?	radio<br>1 Send both testing kits the same day<br>2 Send each testing kit separately at a different time of the year
85	encourage	What might encourage you or make you more likely to try home-based cancer screening in the future: [mark all that apply]	checkbox<br>1 encourage___1 If my partner/spouse also used home based cancer screening<br>2 encourage___2 Recommendation from my doctor<br>3 encourage___3 Talking to someone who has done it before<br>4 encourage___4 Learning more about at-home screening<br>5 encourage___5 Receiving information from my insurance company<br>6 encourage___6 Other (please list below)<br>7 encourage___7 None of the above (nothing would encourage me)<br><br>Field Annotation: @NONEOFTHEABOVE='7'
86	encourage_other<br><br>Show the field ONLY if:<br>[encourage(6)] = '1'	Other:	notes
87	page9		descriptive
88	demo_text	Section Header: *Demographics*<br>In this last section we will ask you some questions about your personal characteristics. This demographic information is used to make sure we hear from people of various backgrounds and walks of life. This is important because we want to make sure that what we learn from this survey reflects the thoughts and feelings of many different communities across the state of Michigan.	descriptive
89	zipcode	What is the zipcode (postal code) where you currently live?	text (zipcode)
90	education	What is the highest level of schooling you completed?	radio

				1 Less than a high school degree<br>2 High school graduate or GED<br>3 Some college<br>4 Graduated from college (e.g. associate or bachelor degree)<br>5 Post graduate degree (e.g. master or doctorate degree)
	91	income	Which of the following best describes your feelings about your current household income?	radio<br>1 Living comfortably on current income<br>2 Getting by on current income<br>3 Finding it difficult on current income<br>4 Finding it very difficult on current income
	92	race	Which of the following best describes your race? [Mark all that apply]	checkbox<br>1 race___1 Asian<br>2 race___2 American Indian or Alaska Native<br>3 race___3 Black or African American<br>4 race___4 Middle Eastern or North African<br>5 race___5 Native Hawaiian or Other Pacific Islander<br>6 race___6 White or Caucasian<br>7 race___7 Some other race or origin (please list below)
	93	race_other<br>Show the field ONLY if:<br>[race(7)] = '1'	Some other race or origin:	text
	94	ethnicity	Are you of Hispanic, Latino, or Spanish ethnicity?	radio<br>1 Yes<br>2 No
	95	relationship	Which of the following best describes your current relationship status?	radio<br>1 Married or partnered<br>2 Single, never married or partnered<br>3 Widowed<br>4 Divorced<br>5 Separated
	96	health_insurance	Which of the following best describes your primary source of insurance/health care coverage?	radio<br>1 A plan you or another family member has through an employer or union<br>2 A plan that you or another family member buys on your own<br>3 Medicaid (Healthy Michigan or other state program)<br>4 Medicare<br>5 TRICARE (formerly CHAMPUS), VA, or Military<br>6 Alaska Native, Indian Health Service, Tribal Health Service<br>7 Another form of insurance/healthcare coverage

				8 No insurance/healthcare coverage
	97	disability	Do you identify as someone with a physical disability or mobility impairment?	radio<br>1 Yes<br>2 No
	98	survey	How did you hear about this survey?	radio<br>1 From my healthcare provider<br>2 From a community newsletter<br>3 From a Facebook post or ad<br>4 From the University of Michigan Health Research website<br>5 From a family member or friend<br>6 From some other source (please list below)
	99	survey_other<br>Show the field ONLY if:<br>[survey] = '6'	From some other source:	notes
	100	free_response	Is there anything else that we haven't asked about that you think would be important for us to know?	notes
	101	page10		descriptive
	102	english_survey_complete	Section Header: *Form Status*<br>Complete?	dropdown<br>0 Incomplete<br>1 Unverified<br>2 Complete
